# Genome Sequencing and Analysis of the Postharvest Fungus *Penicillium expansum* R21

**DOI:** 10.1128/genomeA.01516-16

**Published:** 2017-02-16

**Authors:** Guohua Yin, Yuliang Zhang, Sui Sheng T. Hua, Jiujiang Yu, Lijing Bu, Kayla K. Pennerman, Qixing Huang, Anping Guo, Joan W. Bennett

**Affiliations:** a Key Laboratory of Biology and Genetic Resources of Tropical Crops, Ministry of Agriculture, Institute of Tropical Bioscience and Biotechnology, Chinese Academy of Tropical Agricultural Sciences, Haikou, Hainan, China; b Department of Plant Biology and Pathology, Rutgers, the State University of New Jersey, New Brunswick, New Jersey, USA; c Department of Agriculture, ARS, Western Regional Research Center, Albany, California, USA; d Department of Agriculture, ARS, Beltsville Agricultural Research Center, Beltsville, Maryland, USA; e Department of Biology, Center for Evolutionary & Theoretical Immunology (CETI), University of New Mexico, Albuquerque, New Mexico, USA

## Abstract

Blue mold is the vernacular name of a common postharvest disease of stored apples, pears, and quince that is caused by several common species of *Penicillium*. This study reports the draft genome sequence of *Penicillium expansum* strain R21, which was isolated from a red delicious apple in 2011 in Pennsylvania.

## GENOME ANNOUNCEMENT

*Penicillium expansum* causes an economically serious postharvest decay of fruits and is known as “blue mold” in reference to the abundant blue-green spores produced by this species and several other *Penicillium* species that cause the disease (1, 2). *P. expansum* also produces patulin, a mycotoxin that can contaminate apple juice and other apple products which is regulated by the US Food and Drug Administration, the European Union, and other regulatory agencies (3). The Food and Agriculture Organization of the United Nations has reported that about one fourth of the world’s food crops are contaminated with mycotoxins every year (4, 5). Very little is known about the genetic basis of the infection, pathogenicity, toxigenicity, and virulence of penicillia. In order to explore the possible fungal virulence factors and to devise novel strategies for the mitigation of mycotoxin contamination in fruits, our group has sequenced the genomes of three related *Penicillium* species (6, 7, 8). Here, we report the genome of the wild-type *P. expansum* strain R21, which produces patulin during fruit infection.

Spores of *P. expansum* R21 isolated in 2011 from a decomposing red delicious apple in Pennsylvania were inoculated in potato dextrose broth and incubated with shaking at 200 rpm at 25°C for 7 days. Total genomic DNA was extracted using an E.Z.N.A. Fungal DNA midi kit (Omega Bio-Tek) according to the manufacturer’s instructions. Three DNA libraries with different inserted size (paired-end 410-bp, mate pair 2kbp+8kbp) of *P. expansum* R21 were sequenced using an Illumina MiSeq benchtop sequencer and the sequence depths reached 105×, 109×, and 84×, respectively. *De novo* assembly was performed on all data using SOAPdenovo v2.04 (http://soap.genomics.org.cn) with 59 k-mer after a k-mer sweep evaluation. The contigs was then extended using reference guided assembly improvement tool AlignGraph (https://github.com/baoe/AlignGraph) with one *P. expansum* reference genome (ASM76974v1) and scaffolding by SSPACE-Standard (9). The final assembly consisted of 573 contigs, with an *N*_50_ value of 107,307 bp. Based on the 17 k-mer statistical analysis, the estimated genome size of *P. expansum* R21 was 34 Mb. The G+C content of the genome was ~48%. Our new assembly has total genome size of 35,046,069 bp, with total number of 187,101 Ns, and 34,858,968 bases without N.

The genome sequence of *P. expanum* R21 has been annotated using the MAKER2 program (10). Annotation results indicate that the *P. expansum* R21 contains 12,707 predicted genes, with an average length of 1,478 bp. The gene total length (all the coding sequences) is 18,790,875 bp, which is 54% of the whole genome. Approximately 282 kb of repeated regions were found, accounting for 0.80% of the genome size. The genomic data will provide useful information for better understanding of how to control blue rot contamination caused by *P. expansum*.

### Accession number(s).

This whole-genome shotgun project has been deposited at DDBJ/ENA/GenBank under the accession number MJGF00000000. The version described in this paper is version MJGF02000000.
